# Blood cells and hematological parameters of Chiala Mountain Salamander, *Batrachuperus karlschmidti* (Urodela, Hynobiidae)

**DOI:** 10.7717/peerj.15446

**Published:** 2023-05-19

**Authors:** Xiuying Liu, Zhangqiang You, Wei Luo, Jianli Xiong, Guangli Wang

**Affiliations:** 1 School of Resources and Environmental Engineering, Mianyang Normal University, Mianyang, Sichuan, China; 2 Ecological Security and Protection Key Laboratory of Sichuan Province, Mianyang Normal University, Mianyanng, Sichuan, China; 3 Xingtai University, Xingtai, Hebei, China

**Keywords:** Blood, Mountain salamander, Hematological characteristics, Sexual difference, Physiological adaptation

## Abstract

Hematological parameters are essential indices for assessing the function of blood and reflecting not only the health status of animal but also their physiological adaptation to the environment. Herein, the composition of blood cells and the hematological parameters of wild *Batrachuperus karlschmidti* were examined for the first time, and the effects of sex, body size, body mass, and age on the hematological parameters were explored. The morphology and morphometric data of the blood cells, as well as the hematological parameters, of *B. karlschmidti* were slightly differ from those of its congener. However, hematological differences between sexes were only found in erythrocyte and leukocyte count, and mean cell volume (MCV), which possibly reflecting the need for better oxygen distribution and stronger immune protection for reproduction. Hematocrit (Hct) and mean cell hemoglobin (MCH) were strongly dependent on body mass. These also might have been attributed to higher oxygen requirements with larger body masses. This is a pilot project exploring the hematology of this species that may help establish hematological parameters in future for supporting species protection and monitoring studies, as well as help understanding the physiological adaptation of this species.

## Introduction

The peripheral blood of vertebrates comprises a variety of different types of cells, including erythrocytes, leukocytes, and thrombocytes, that provide specific and unique functions to the organism. Erythrocytes function to carry oxygen and carbon dioxide throughout the body (Allender & Fry, 2008; Kholiqov et al., 2020; Pernow et al., 2019; Thorn et al., 2020; Vaissi et al., 2012; Xiong et al., 2018a); leukocytes function to protect the body against infection by foreign invaders and prevent disease (Xiong et al., 2018a; Davis, Maney & Maerz, 2008), and thrombocytes regulate hemostasis, coagulation, inflammation and immunity (Vieira-de-Abreu et al., 2012; Arikan & Çiçek, 2014). Blood plasma consists of water, nutritional molecules derived from food, hormones, antibodies, enzymes, and wastes products from the breakdown of enzymes and other metabolized molecules (Seiverd, 1972). Maintaining the composition and levels of each of the components in blood is essential for ensuring homeostasis and proper health animals (Vieira-de-Abreu et al., 2012).

Hematology refers to the study of blood and its related diseases (Seiverd, 1972). The hematological parameters of blood are of significant clinical relevance, as deviations from normal values can be indicative of certain diseases. In peripheral blood, the most common hematological parameters are hemoglobin (Hb) concentration, erythrocyte count, leukocyte count, hematocrit (Hct), erythrocyte mean cell volume (MCV), mean cell hemoglobin (MCH), mean cell hemoglobin concentration (MCHC), and differential leukocyte count (Xiong et al., 2017). Even within a particular species, these parameters can vary depending on both external (*e.g*., temperature, altitude, season, and stress) and internal (*e.g.*, physiological and pathological conditions) environments (Vieira-de-Abreu et al., 2012; Sacchi et al., 2020; Eatwell, Hedley & Barron, 2014; Davis, 2008). Furthermore, some authors (Parrino et al., 2018) have highlighted the effects of anthropogenic factors on hematological parameters. Thus, hematological parameters have been widely used to assess the health status of animals and their physiological adaptation to the environment (Davis, 2008; Hidalgo-Vila et al., 2007; Forzan et al., 2017; D’Amico et al., 2016; Barajas-Valero et al., 2021).

The Chiala mountain salamander (*Batrachuperus karlschmidti*) is a hynobiid species endemic to China. *B. karlschmidti* lives in cold water and can be found under stones within small mountain streams (Liu, 1950). This species is mainly distributed throughout the mountains of Western Sichuan Province (Fu & Zeng, 2008; Lu et al., 2012; Xia et al., 2012; Xiong, Luo & Zeng, 2020) and Southeastern Gansu Province (Ding et al., 2014), and it is listed as vulnerable in the Red List of China’s Vertebrates (Jiang et al., 2016). To date, only the genetic diversity (Fu et al., 2001), skull morphometry (Liu et al., 2020), and phylogeny (Lu et al., 2012; Xia et al., 2012; Fu et al., 2001) of this species have been studied, while no information on its physiology or hematology has been reported. Therefore, this study aimed to characterize the morphologies of the blood cells, as well as the hematological parameters of *B. karlschmidti* and expore the influence of sex, body size, body mass, and age on the hematological characteristics of the organism. These parameters were determined based on wild specimens of *B. karlschmidti* obtained from Xinduqiao in Sichuan Province. This is a pilot project exploring the hematology of this species that may help establish hematological parameters in future for supporting species protection and monitoring studies, as well as help understanding the physiological adaptation of this species.

## Material and Methods

A total of 20 adult salamanders (12 females and 8 males) were collected by hand from Xinduqiao (29°47′26.11″N, 101°33′44.12″E, 3,743 m a.s.l.) in Sichuan Province, China, in June of 2016. Based on external appearance, all specimens were healthy. After the animals were euthanized with MS-222 (Yu’anbao, Shandong Jinjiang Shui’an Biotechnology Co Ltd., Jinan, SHG, China), the snout-vent length (SVL, body size) and body mass of each specimen were measured using a digital caliper (Chixi, Chixi Corp., Shanghai, China, with an accuracy of 0.01 mm) and electronic scale (Yingheng, Yingheng Corp., Shanghai, China, with an accuracy of 0.01 g), respectively. Then, the animals were dissected to expose the heart for collecting blood samples from aortic arch using heparinized hematocrit capillaries within 1~2 min after euthanized. The longest phalange of the left hindlimb of each specimen was excised for age determination by skeletochronology method used by Xiong et al. (2020). In brief, phalanges were washed with tap water, decalcified in Plank decalcifying solution, dehydrated in alcohol, after treated using n-butyl alcohol, embedded in paraffin. The embedded phalanges were sliced using a Leica RM 2135 type microtome (Leica Microsystems, Wetzlar, Germany), then the slices were stained with Haematoxylin-Eosin (HE). Age was determined by counting the number of line of arrested growth (LAG) in the periosteal bone of the phalanges under a light microscope (Olympus CX31; Olympus, Tokyo, Japan). The sex of the salamander was determined *via* inspection of the gonads. Animal samples were stored in 10% formalin and deposited in Museum of Mianyang Normal University.

Blood smears were prepared using slide on slide wedge technique (Eatwell, Hedley & Barron, 2014) to examine the morphology and morphometry of the blood cells. Dried blood smears were stained with Wright’s stain and observed under a light microscope (Olympus CX31; Olympus, Tokyo, Japan). For the morphometric characterization of the cell size, the erythrocyte length (EL), erythrocyte width (EW), nucleus length (NL), and nucleus width (NW) of 50 erythrocytes that were randomly extracted per individual, were measured using the BioLife Std microscopic image analysis software (Beijing iCALIBUR Research & Development Center). Erythrocyte area (EA) and nucleus area (NA) were calculated according to the formulae EA = ELEWπ/4 and NA = NLNWπ/4, respectively (Arikan & Çiçek, 2014). The shapes of the erythrocyte and erythrocyte nucleus were calculated according to the EL/EW and NL/NW ratios (Tok et al., 2009). Hemoglobin (Hb) concentration was measured using Sahli hemometer, and the hematocrit (Hct) was calculated from the proportion of the blood cell volume in the total blood volume after standard centrifugation (Xiangzhi Centrifuge TG12; Changsha Xiangzhi Centrifuge Instrument Co Ltd, relative centrifugation force: 1,4800 g) in microhematocrit tubes at 12, 000 pm for 5 min. Mean cell volume (MCV), mean cell hemoglobin (MCH), and mean cell hemoglobin concentration (MCHC) were all calculated using Wintrobe’s formula (Wintrobe, 1933). The blood was diluted 20 times by saline solution, then erythrocyte count was determined manually using a Neubauer hemocytometer under a light microscope (OLYMPUS CX31; Olympus, Tokyo, Japan), while the leukocyte count was determined as the proportion of erythrocytes to leukocytes in 10 random fields as described by Xiong et al. (2017, 2018a, 2018b). A total of 100 leukocytes were counted in the blood smear of each specimen to determine the differential leukocyte count, which was manually performed under a light microscope (OLYMPUS CX31; Olympus, Tokyo, Japan). Then, the percentage of each leukocyte type for each individual was calculated according to the observed data. Cell photomicrographs were taken with a mounted OLYMPUS DP26 digital camera.

Prior to analysis, leukocyte differential count was transformed by centred log-ratio transformation (clr) using CoDaPack v2.02.21 (Comas & Thiò-Henestrosa, 2011) as it is a composition data (Aitchison, 1982). The erythrocyte morphometry, differential leukocyte count, and hematological parameters were tested for normality using the Kolmogorov-Smirnov test. To compare the difference between sexes, independent sample t-tests were conducted to analyze the normally distributed variables, while the Mann-Whitney U-test was employed for analyzing the non-normally distributed variables. Linear regression was used to determine the relationships between erythrocyte morphometry and hematological parameters and the SVL, body mass, and age. All statistical tests were performed using the SPSS software package (SPSS Inc., Chicago, IL, USA, Version 22.0). All data were presented as the mean ± SE, and the significance level used in all tests was *P* < 0.05.

All experiments were carried out according to protocols approved by the Animal Care and Use Committee of Ecological Security and Protection Key Laboratory of Sichuan Province, Mianyang Normal University (MYNU-ESPKL201605007).

## Results

**Figure 1:**
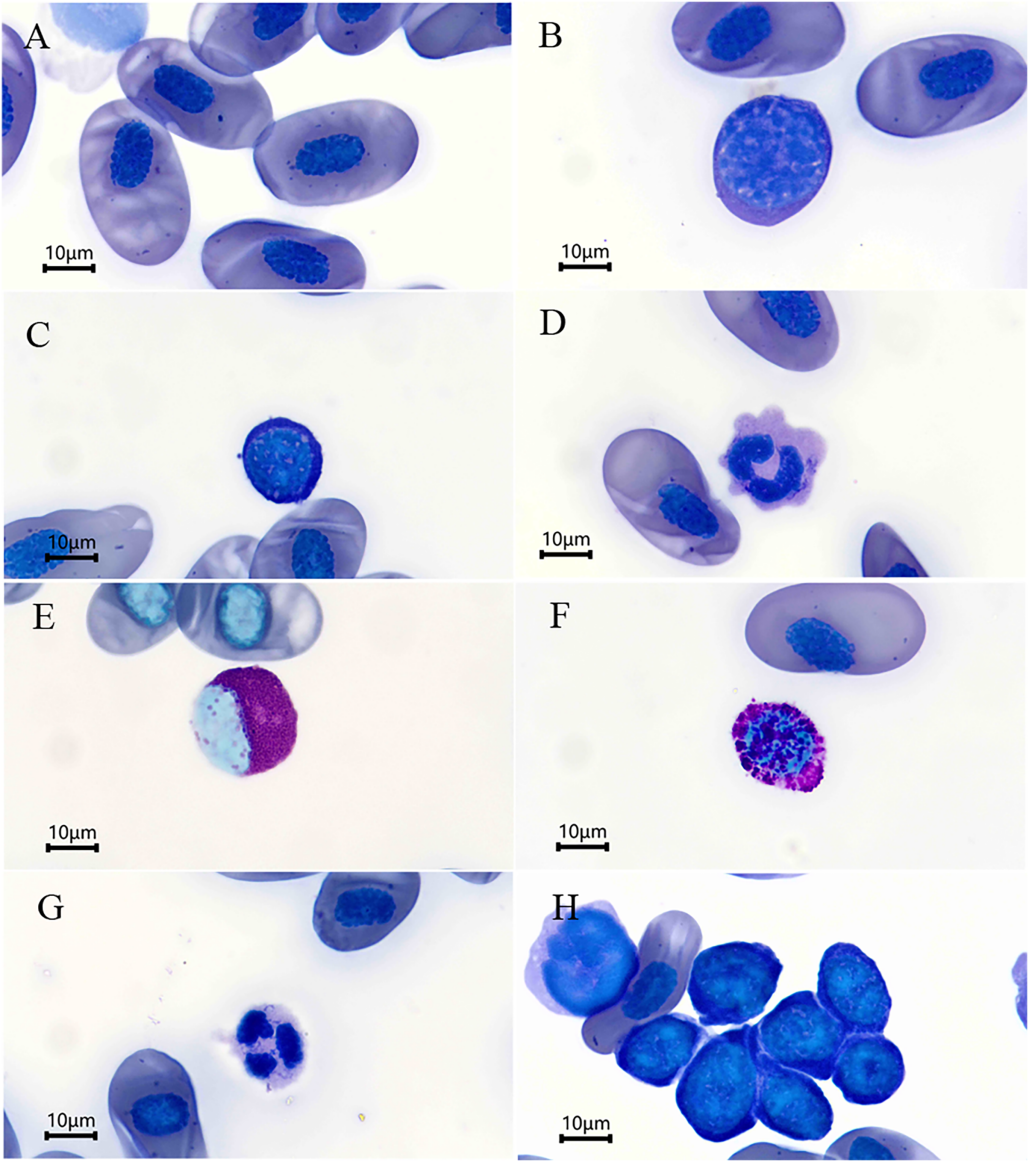
Blood cells (Wright’s stain) of *Batrachuperus karlschmidti*. (A) Erythrocyte, (B) large lymphocytes, (C) small lymphocytes, (D) monocyte, (E) eosinophil, (F) basophil, (G) neutrophil, (H) a group of thrombocytes.

The morphological results of the blood cells are depicted in Fig. 1. The erythrocytes were ovular in shape with an ellipsoidal nucleus located either at the center of the erythrocyte or off-center (Fig. 1A). The erythrocyte morphometric data are summarized in Table 1. Except EL/EW and NL/NW, the female salamanders had larger mean morphometric data than the males; however, the independent sample t-tests revealed that there were no statistical significantly differences in the morphometric parameters evaluated between sexes (*P* > 0.05 for all, Table 1).

**Table 1:** Morphometric data of erythrocytes in *Batrachuperus karlschmidti*, and the results of comparison between males and females using independent sample t-tests.

Characters	Sex	Mean ± SE	Range	Sig
	♀	35.66 ± 0.60	32.85–39.07	
Erythrocyte length (EL, μm)	♂	35.51 ± 0.63	33.22–38.51	*P* = 0.867
	♀♂	35.60 ± 0.43	32.85–39.07	
	♀	21.79 ± 0.34	19.43–24.15	
Erythrocyte width (EW, μm)	♂	21.22 ± 0.41	19.60–22.61	*P* = 0.304
	♀♂	21.56 ± 0.26	19.43–24.15	
	♀	610.52 ± 16.55	528.75–714.48	
Erythrocyte size (EA, μm^2^)	♂	591.75 ± 17.09	511.02–664.75	*P* = 0.456
	♀♂	603.01 ± 11.94	511.02–714.47	
	♀	1.64 ± 0.03	1.47–1.81	
EL/EW	♂	1.68 ± 0.04	1.51–1.83	*P* = 0.441
	♀♂	1.65 ± 0.02	1.47–1.83	
	♀	15.23 ± 0.33	13.44–17.48	
Nucleus length (NL, μm)	♂	15.08 ± 0.21	14.16–15.93	*P* = 0.729
	♀♂	15.17 ± 0.21	13.44–17.48	
	♀	9.58 ± 0.18	8.61–10.42	
Nucleus width (NW, μm)	♂	9.19 ± 0.16	8.23–9.57	*P* = 0.139
	♀♂	9.43 ± 0.13	8.23–10.42	
	♀	114.99 ± 4.43	90.84–142.99	
Nucleus size (NA, μm^2^)	♂	108.89 ± 2.97	91.52–117.73	*P* = 0.321
	♀♂	112.55 ± 2.93	90.84–142.99	
	♀	1.59 ± 0.02	1.51–1.68	
NL/NW	♂	1.64 ± 0.02	1.53–1.72	*P* = 0.066
	♀♂	1.61 ± 0.01	1.51–1.72	

**Note:**

EL/EW, the ratio of erythrocyte length (EL) and erythrocyte width (EW); NL/NW, the ratio of nucleus length (NL) and nucleus width (NW).

The leukocytes identified were divided into lymphocytes, monocytes, eosinophils, basophils, and neutrophils according to their morphological characteristics (Figs. 1B–1G). The lymphocytes were round or slightly elliptical in shape and contained a small amount of cytoplasm, and they were further divided into large and small lymphocytes according to their size (Figs. 1B and 1C). The monocytes were similar in size to the large lymphocytes, but their nuclei were round, kidney-shaped, or horseshoe-shaped and occupied at least one-half two two-thirds the volume of the cells (Fig. 1D). The eosinophils were characterized by the presence of some visibly large, reddish, shiny granules in the cytoplasm, and their nuclei was lobed or bilobed (Fig. 1E). The basophils were characterized by the presence of round, lightly basophilic granules of various sizes throughout the nucleus and the entire cell (Fig. 1F). Lastly, the neutrophils were spherical in shape with a multiple-lobed or segmented nucleus (Fig. 1G). The nucleus of thrombocytes was round to oval, and filled almost the whole cell. Thrombocytes tended to clump together in blood smears (Fig. 1H). The percentages of each type of leukocyte are shown in Table 2. The lymphocytes were the most abundant leukocyte in the blood smears, and followed by monocytes, basophils, neutrophils, and eosinophils in descending order. There was no significant difference in the abundance of each type of leukocyte between sexes (*P* > 0.05 for all, Table 2).

**Table 2:** Differential leukocytes count observed in *Batrachuperus karlschmidti*, and the results of comparison between males and females using independent sample t-tests or Mann-Whitney U-test.

Characters	Sex	Mean ± SE	Range	Sig
	♀	82.17 ± 1.63	70–90	
Lymphocytes (%)	♂	82.75 ± 1.58	77–88	*P* = 0.938*
	♀♂	82.40 ± 1.14	70–90	
	♀	6.67 ± 1.00	2–14	
Monocytes (%)	♂	6.50 ± 1.05	4–13	*P* = 0.804*
	♀♂	6.60 ± 0.72	2–14	
	♀	5.00 ± 0.79	1–9	
Basophils (%)	♂	5.00 ± 0.60	2–7	*P* = 0.970^#^
	♀♂	5.00 ± 0.52	1–9	
	♀	3.75 ± 0.61	1–7	
Neutrophils (%)	♂	3.88 ± 0.52	2–6	*P* = 0.678^#^
	♀♂	3.80 ± 0.41	1–7	
	♀	2.42 ± 0.23	1–4	
Eosinophils (%)	♂	1.88 ± 0.40	1–4	*P* = 0.083*
	♀♂	2.20 ± 0.21	1–4	

**Notes:**

* Independent sample t-tests.

# Mann-Whitney U-test.

The hematological parameters of *B. karlschmidti* are shown in Table 3. Except MCV and MCH, males had larger mean values for all hematological parameters than females, but the differences in erythrocyte count (*P* = 0.001), leukocytes count (*P* = 0.002), and MCV (*P* = 0.016) were the only parameters that were statistically significant between sexes.

**Table 3:** Hematological parameters observed in *Batrachuperus karlschmidti*, and the results of comparison between males and females using independent sample t-tests or Mann-Whitney U-test.

Characters	Sex	Mean ± SE	Range	Sig
	♀	3.42 ± 0.51	1.5–7.0	
Hemoglobin (g/dl)	♂	4.81 ± 0.85	2.0–8.5	*P* = 0.150*
	♀♂	3.98 ± 0.47	1.5–8.5	
	♀	5.50 ± 0.56	3.50–10.00	
Erythrocyte count (10^4^/mm^3^)	♂	10.44 ± 1.28	2.50–14.50	*P* = 0.001*
	♀♂	7.48 ± 0.81	2.50–14.50	
	♀	1.35 ± 0.16	0.72–2.40	
Leukocyte count (10^3^/mm^3^)	♂	3.06 ± 0.37	1.79–4.40	*P* = 0.002*
	♀♂	2.04 ± 0.26	0.72–4.40	
	♀	29.88 ± 2.83	20.55–52.95	
Hct (%)	♂	32.83 ± 3.85	17.95–46.05	*P* = 0.536*
	♀♂	31.06 ± 2.25	17.95–52.95	
	♀	5.76 ± 0.48	2.27–8.46	
MCV (10^3^/fl)	♂	3.61 ± 0.68	2.11–8.00	*P* = 0.016*
	♀♂	4.90 ± 0.46	2.11–8.46	
	♀	0.62 ± 0.07	0.35–1.08	
MCH (10^3^/pg)	♂	0.49 ± 0.07	0.22–0.80	*P* = 0.238^#^
	♀♂	0.57 ± 0.05	0.22–1.08	
	♀	11.43 ± 1.17	5.07–15.95	
MCHC (%)	♂	14.37 ± 1.49	8.24–20.21	*P* = 0.134*
	♀♂	12.61 ± 0.95	5.07–20.21	

**Notes:**

* Independent sample t-tests.

# Mann-Whitney U-test.

Hct, hematocrit; MCV, mean cell volume; MCH, mean cell hemoglobin; MCHC, mean cell hemoglobin concentration.

In females, the mean SVL, body mass, and ages were determined to be 83.97 ± 2.11 mm (range: 70.36–96.42), 14.15 ± 1.64 g (range: 8.26–28.16), and 8.50 ± 0.70 years (range: 5–13), respectively, while these values in males were 79.57 ± 2.08 mm (range: 65.97–83.53), 13.61 ± 1.24 g (range: 9.15–19.53), and 8.38 ± 1.03 years (range: 4–13), respectively. Based on these results, females had larger mean SVL and body mass and were older than the males. Results of linear regression showed that only Hct (*r* = 0.500) and MCH (*r* = 0.631) were significantly affected by body mass (Table 4).

**Table 4:** Influence of SVL, body mass, and age on the erythrocyte morphometry and hematological parameters in *Batrachuperus karlschmidti* using linear regression.

Characters	Snout-vent length	Body mass	Age
Erythrocyte length	F_1,18_ = 0.532, *P* = 0.475	F_1,18_ = 1.053, *P* = 0.318	F_1,18_ = 1.516, *P* = 0.234
Erythrocyte width	F_1,18_ = 0.000, *P* = 0.997	F_1,18_ = 0.900, *P* = 0.355	F_1,18_ = 0.224, *P* = 0.641
Erythrocyte size	F_1,18_ = 0.201, *P* = 0.659	F_1,18_ = 1.525, *P* = 0.223	F_1,18_ = 1.179, *P* = 0.292
EL/EW	F_1,18_ = 0.315, *P* = 0.582	F_1,18_ = 0.002, *P* = 0.964	F_1,18_ = 0.374, *P* = 0.548
Nucleus length	F_1,18_ = 0.000, *P* = 0.989	F_1,18_ = 0.284 *P* = 0.600	F_1,18_ = 1.603, *P* = 0.222
Nucleus width	F_1,18_ = 0.015, *P* = 0.904	F_1,18_ = 0.000, *P* = 0.991	F_1,18_ = 1.329, *P* = 0.264
Nucleus size	F_1,18_ = 0.007, *P* = 0.936	F_1,18_ = 0.084, *P* = 0.776	F_1,18_ = 1.425, *P* = 0.248
NL/NW	F_1,18_ = 0.038, *P* = 0.847	F_1,18_ = 0.742, *P* = 0.400	F_1,18_ = 0.026, *P* = 0.873
Hemoglobin	F_1,18_ = 0.038, *P* = 0.848	F_1,18_ = 4.353, *P* = 0.051	F_1,18_ = 2.130, *P* = 0.162
Erythrocyte count	F_1,18_ = 0.761, *P* = 0.394	F_1,18_ = 0.046, *P* = 0.832	F_1,18_ = 1.386, *P* = 0.254
Leucocyte count	F_1,18_ = 1.059, *P* = 0.317	F_1,18_ = 1.332, *P* = 0.264	F_1,18_ = 0.037, *P* = 0.849
Hct	F_1,18_ = 2.250, *P* = 0.151	F_1,18_ = 5.998, *P* = 0.025	F_1,18_ = 1.386, *P* = 0.254
MCV	F_1,18_ = 0.685, *P* = 0.419	F_1,18_ = 3.296, *P* = 0.086	F_1,18_ = 0.731, *P* = 0.404
MCH	F_1,18_ = 0.853, *P* = 0.368	F_1,18_ = 11.928, *P* = 0.003	F_1,18_ = 0.631, *P* = 0.438
MCHC	F_1,18_ = 2.340, *P* = 0.143	F_1,18_ = 0.328, *P* = 0.574	F_1,18_ = 1.608, *P* = 0.221

**Note:**

EL/EW, the ratio of erythrocyte length (EL) and erythrocyte width (EW); NL/NW, the ratio of nucleus length (NL) and nucleus width (NW); Hct, hematocrit; MCV, mean cell volume; MCH, mean cell hemoglobin; MCHC, mean cell hemoglobin concentration.

## Discussion

This study reported the morphologies, composition, and morphometry of various types of cells in the blood, as well as the hematological parameters, of wild *B. karlschmidti*. The differences in these hematological characteristics, as well as the relationships between erythrocytes morphometry, hematological parameters and SVL, body mass, and age were also elucidated. The results indicated that erythrocyte and leukocyte count, and MCV showed significant sexual difference, Hct and MCH were positive correlate with body mass.

The *Batrachuperus* genus of salamanders is endemic to China, in which six species have been recognized to date (Frost, 2022). These species are primarily distributed throughout the mountainous areas of Western China. At present, only the hematological properties of *B. tibetanus* (Huang, Zhang & Wang, 2004; Xiong et al., 2022), *B. pinchonii* (Xiong et al., 2018a), *B. yenyuanensis* (Xiong et al., 2017), *B. londongensis* (Zhang et al., 2018) have been reported. In the blood of *B. karlschmidti*, the erythrocytes and leukocytes, of which five different cell types were identified, featured the morphologies similar to those previously reported not only in other species of the genus *Batrachuperus* but also other amphibians, indicating that amphibians have morphologically similar blood cells (Meesawat, Kitana & Kitana, 2016). However, the hematological parameters and the composition of the five types of leukocytes varied among species, which might be indicative of species-specific characteristics (Table 5).

**Table 5:** Morphometric data of erythrocyte, hematological parameters, and differential leukocyte counts in genus *Batrachuperus*.

Characters	*B. karlschmidti*	*B. pinchonii* ^a^		*B. londongensis* ^b^	*B. yenyuanensis* ^c^	*B. tibetanus*			
Population	Xinduqiao	Jiajin	Sandaoping	Wawushan	Yanyuan	Zhouzhi^d^	Meixian^e^	Taibai^e^	Xihe^e^
Erythrocyte length (EL, μm)	35.60	36.13	37.67	–	36.40	–	37.34	36.45	34.34
Erythrocyte width (EW, μm)	21.56	21.41	21.67	–	22.99	–	19.05	19.44	18.87
Erythrocyte size (EA, μm^2^)	603.01	607.07	637.44	–	840.39	–	559.78	557.06	510.10
EL/EW	1.65	1.69	1.76	–	1.58	–	–	–	–
Nucleus length (NL, μm)	15.17	15.11	15.41	–	15.25	–	13.64	13.20	13.16
Nucleus width (NW, μm)	9.43	8.97	9.11	–	9.77	–	7.15	7.06	6.80
Nucleus size (NA, μm^2^)	112.55	106.51	110.47	–	149.58	–	76.89	73.31	70.55
NL/NW	1.61	1.69	1.69	–	1.57	–	–	–	–
Hemoglobin (g/dl)	3.98	5.02	5.38	4.16	5.00	–	6.27	5.73	4.38
Erythrocyte count (10^4^/mm^3^)	7.48	7.00	7.42	6.04	7.20	7.80	7.41	7.17	5.99
Leukocyte count (10^3^/mm^3^)	2.04	2.46	2.49	2.90	3.10	3.40	3.09	3.43	4.45
Hct (%)	31.06	–	–	–	30.49	–	29.85	29.39	23.62
MCV (10^3^/fl)	4.90	–	–	–	4.57	–	4.12	4.27	4.22
MCH (10^3^/pg)	0.57	–	–	–	0.75	–	0.88	0.83	0.76
MCHC (%)	12.61	–	–	–	16.61	–	21.97	20.75	18.75
Lymphocytes (%)	82.40	60.22	67.16	80.26	82.73	–	–	–	–
Monocytes (%)	6.60	12.22	9.96	6.08	6.55	–	–	–	–
Basophils (%)	5.00	2.28	2.48	4.59	3.04	–	–	–	–
Neutrophils (%)	3.80	6.61	5.28	4.44	4.18	–	–	–	–
Eosinophils (%)	2.20	18.72	15.12	3.37	3.51	–	–	–	–

**Notes:**

a Data from Xiong et al. (2018a).

b Data from Zhang et al. (2018).

c Data from Xiong et al. (2017).

d Data from Huang, Zhang & Wang (2004).

e Data from Xiong et al. (2022).

EL/EW, the ratio of erythrocyte length (EL) and erythrocyte width (EW); NL/NW, the ratio of nucleus length (NL) and nucleus width (NW); Hct, hematocrit; MCV, mean cell volume; MCH, mean cell hemoglobin; MCHC, mean cell hemoglobin concentration.

Differences in morphology, behavior, life history, and physiology between sexes are common in the animal kingdom. Differences in certain hematological characteristics between sexes have also been widely discovered in Urodela. For example, leukocyte count in *Lyciasalamandra fazilae* (Tok et al., 2009), erythrocyte size and shape in O*mmatotriton ophryticus* (Tosunoğlu et al., 2011), Hct and Hb in *Ambystoma maculatum* (Finkler, 2013), the percentage of neutrophils in *B. yenyuanensis* (Xiong et al., 2017), composition of neutrophils and basophils in *B. londongensis* (Zhang et al., 2018), and erythrocyte count in *B. pinchonii* (Xiong et al., 2018a), all have been reported to vary between sexes. These studies demonstrated that the hematological characteristics of males and females were different in multiple different species of salamanders. In this study, male *B. karlschmidti* had significant higher erythrocyte and leukocyte counts but significant lower MCV than female. The erythrocyte count reflects the capacity of the organism to carry oxygen throughout the body, meaning a greater number of erythrocytes count can enable a greater capacity to distribute oxygen (Xiong et al., 2018a, 2017; Davis, 2008). Leukocytes are the important components of the immune system, so the leukocyte count is one of the most common parameters used to assess the cellular immune response and health (Davis, 2008). A higher leukocyte count typically translates to a more robust immune response (Fokidis, Greiner & Deviche, 2008), which can help to mitigate the risk of infection and disease development. MCV is also related to the capacity to carry oxygen. A lower MCV is reflective of a reduced consumption of oxygen by erythrocytes and, therefore, better transport of oxygen (Bai et al., 2021).

During the reproductive season, male hynobiid salamanders, such as *Hynobius nigrescens* (Hasumi, 1994), *H. guabangshanensis* (Guo, Mi & Deng, 2008), and *H. leechii* (Park, Park & Yang, 1996; Park & Park, 2000), undergo significant and costly metabolic changes to attract mates as well as scramble competition to fertilize the eggs (Xiong et al., 2018a). These activities increase male susceptibility to infection (Klein, 2000), while would require greater levels of oxygen, as well as higher oxygen carrying (Tosunoğlu et al., 2011), and higher immune capacities, than normal physiological functions to fight the infection. The *B. karlschmidti* specimens used in this study were collected in June, which is during their reproductive season (Liu, 1950). So far, there are no reports concerning the reproductive biology of *B. karlschmidti*. We speculated that the higher erythrocyte and leukocyte counts, and lower MCVs in male *B. karlschmidti* might be related to the reproductive behavior of males and, might be reflective of the need for greater oxygen carrying capacities and stronger immune responses to facilitate reproduction.

Snout-vent length, body mass, and age can affect many functions in animals, such as respiration, metabolism, locomotion, and digestion. However, the effects of SVL, body mass, and age on the hematological characteristics vary among Urodela species. For example, SVL positively related to the dimensions of erythrocytes in *Ambystoma talpoideum* (Davis, 2008) and *Plethodon cicnereus* (Maerz et al., 2009), while SVL and body mass had no correlation with any of the hematological parameters of *B. londongensis* (Zhang et al., 2018). In this study, among SVL, body mass, and age, only body mass had a statistically significant positive correlated to Hct and MCH of *B. karlschmidti*. Hct and MCH are a representation of organism’s oxygen carrying capacity, such that higher Hct and MCH values indicate higher oxygen carrying capacities. Oxygen requirements also increase with increases in body size and body mass (Feder, 1976, 1977). Thus, higher Hct and MCH values were representative of higher oxygen requirements due to higher body mass.

## Conclusions

To conclude, this was the first study to analyze the blood cell composition and hematological parameters of *B. karlschmidti*, in which not only the morphologies and morphometric of the blood cells and hematological parameters were investigated, but also the effects of sex, body size, body mass, and age on these hematological characteristics were explored. This is a pilot project exploring the hematology of this species that may help establish hematological parameters in future for supporting species protection and monitoring studies, as well as help understanding the physiological adaptation of this species.

## Supplemental Information

10.7717/peerj.15446/supp-1 Supplemental Information 1 Raw data.

## Additional Information and Declarations

### Field Study Permissions

The following information was supplied relating to field study approvals (*i.e*., approving body and any reference numbers): Animal Care and Use Committee of Ecological Security and Protection Key Laboratory of Sichuan Province, Mianyang Normal University.

### Data Availability

The following information was supplied regarding data availability: The raw data are available in the Supplemental Files.
